# Pathogenesis of autoimmunity in common variable immunodeficiency

**DOI:** 10.3389/fimmu.2012.00210

**Published:** 2012-07-18

**Authors:** Klaus Warnatz, Reinhard E. Voll

**Affiliations:** ^1^Centre of Chronic Immunodeficiency, University Medical Center Freiburg, University of Freiburg,Freiburg, Germany; ^2^Division of Rheumatology and Clinical Immunology, University Medical Center Freiburg,Freiburg, Germany

**Keywords:** autoimmune cytopenia, autoimmunity, CD21^low^ B cells, common variable immunodeficiency, hypogammaglobulinemia

## Abstract

Common variable immunodeficiency (CVID) presents in up to 25% of patients with autoimmune (AI) manifestations. Given the frequency and early onset in some patients with CVID, AI dysregulation seems to be an integral part of the immunodeficiency. Antibody-mediated AI cytopenias, most often affecting erythrocytes and platelets make up over 50% of these patients. This seems to be distinct from mainly cell-mediated organ-specific autoimmunity. Some patients present like patients with AI lymphoproliferative syndrome. Interestingly, in the majority of patients with AI cytopenias the immunological examination reveals a dysregulated B and T cell homeostasis. These phenotypic changes are associated with altered signaling through the antigen receptor which may well be a potential risk factor for disturbed immune tolerance as has been seen in STIM1 deficiency. In addition, elevated B cell-activating factor serum levels in CVID patients may contribute to survival of autoreactive B cells. Of all genetic defects associated with CVID certain alterations in TACI, CD19, and CD81 deficiency have most often been associated with AI manifestations. In conclusion, autoimmunity in CVID offers opportunities to gain insights into general mechanisms of human autoimmunity.

Autoimmunity is an integral part of immune dysregulation in a quarter of patients with common variable immunodeficiency (CVID), often presenting as the first manifestation of the disease (Agarwal and Cunningham-Rundles, 2009). In recent years analyses of the immune disturbances have revealed complex dysregulations of the immune system. In parallel, progress in our comprehension of the pathogenesis of connective tissue disorders like systemic lupus erythematosus (SLE) allows for comparison of common roots of human autoimmune (AI) disorders.

This perspective article is an attempt to summarize the factors which contribute to autoimmunity in CVID.

Autoimmune cytopenias are the most common AI manifestations in CVID and the focus of this article. In the context of distinct associated alterations of the cellular immune system AI cytopenias appear to be a separate manifestation from organ-specific autoimmunity in CVID (Boileau et al., 2011; Cheng and Anderson, 2012). The presentation of AI-CVID patients resembles patients with autoimmune lymphoproliferative syndrome (ALPS) with the coincidence of lymphoproliferation and AI cytopenias (Seve et al., 2008; Wehr et al., 2008; Boileau et al., 2011). While none of the cellular markers, such as increased double negative T cells or reduced switched memory B cells, helped to distinguish AI-CVID from FAS-ALPS, increased serum levels of soluble Fas ligand, interleukin (IL) 10, and vitamin B12 allowed a distinction between FAS-ALPS patients and AI-CVID to be made (Rensing-Ehl et al., 2010). None of the tested CVID patients carried a genomic or somatic mutation in FAS, rendering FAS-ALPS a differential diagnosis. Thus, the reason that lymphoproliferation and autoimmunity are seen together in most of the CVID patients remains obscure. Other causes of ALPS and ALPS-related disorders have not been excluded systematically in AI-CVID.

Other immunodeficiencies strongly associated with AI manifestations comprise immune dysregulation, polyendocrinopathy, enteropathy X-linked (IPEX) syndrome, autoimmune polyendocrine syndrome type 1, combined immunodeficiencies (CID) including hypomorphic severe (S)CID variants (Liston et al., 2008), both calcium channelopathies, Wiskott–Aldrich syndrome (WAS), DiGeorge syndrome, Good syndrome, activation-induced deaminase (AID) deficiency, CD25 deficiency, Stat5b deficiency, and cartilage hair dysplasia (Al-Herz et al., 2011).

Most of these immunodeficiencies are associated with (i) disturbed T cell homeostasis, (ii) altered antigen receptor, or (iii) altered cytokine signaling. Aspects relevant in patients with CVID shall be discussed in the following sections.

## DISTURBED T CELL HOMEOSTASIS IN AI-CVID

Disturbed T cell homeostasis is a common contributing factor to the development of autoimmunity in different forms of monogenic primary immunodeficiency disorders (PIDs). Several features of disturbed cell homeostasis are also present in CVID. Lymphopenia affects mostly CD4 T cells and especially naïve CD4 T cells, while CD8 T cells become relatively expanded (Giovannetti et al., 2007). Both CD4 and CD8 T cells are activated as determined by the expression of activation markers and Ki67. Thymic output was decreased, but Ki67 expression was particularly strong in naïve and central memory T cells, suggesting homeostatic proliferation as described for other immunodeficiency models (Cassani et al., 2010). In addition, the Vβ repertoire of CD4 T cells had contracted. These changes are well known to be associated with an increased risk of autoimmunity as previously demonstrated in murine models and human AI disease (Datta and Sarvetnick, 2009).

The severe reduction in naïve CD4 T cells in CVID has been suggested as a criterion for the diagnosis of late-onset CID (LOCID; Malphettes et al., 2009) for resembling the immunological and clinical phenotype of patients with hypomorphic SCID mutations (Liston et al., 2008; Cassani et al., 2010; De Ravin et al., 2010). Interestingly, the association of CD4 lymphopenia in primary immunodeficiency seems to be stronger with granulomatous inflammatory disease than AI cytopenias (Schuetz et al., 2008; Mouillot et al., 2010). IL-7, which has a key role in the expansion of autoreactive T cell clones in the lymphopenic host, was also found to be elevated in a subgroup of CVID patients (Holm et al., 2005). Though increased IL-7 levels were not associated with T cell lymphopenia, they nevertheless correlated with a more frequent incidence of autoimmunity. The regular feedback mechanism of IL-7 regulation seemed to fail in the small group of AI-CVID patients examined. The production of several other cytokines including IL-2, interferon (IFN)-γ, IL-4, and TNFα is altered in some CVID patients, but none of the reported alterations have been examined for their role in eliciting autoimmunity (Fischer et al., 1994; Fritsch et al., 1994; Mullighan et al., 1997). Testing the role of specific cytokines in this setting will be of great interest as it is likely to reveal potential therapeutic targets.

Skewing of CD8 T cells is often more prominent than that of CD4 T cells (Giovannetti et al., 2007). Cytomegalovirus (CMV) causes immunosenescence associated with terminal differentiation of CD8 effector T cells which results in a skewing of the repertoire. In CVID this phenomenon was exaggerated (Kuntz et al., 2011). A chronic viral infection is therefore a potential trigger for the clinical manifestation of AI disease in a disturbed immune system (Marashi et al., 2011).

Selection, activation, and differentiation of T cells in CVID may also be affected by an impaired response of the T cell receptor after stimulation (Fischer et al., 1994; Boncristiano et al., 2000; Paccani et al., 2005). However, to date, the published investigations neither report an underlying genetic defect nor a correlation between altered T cell receptor signaling and a higher prevalence of autoimmunity. Currently, the only intrinsic T cell defect which causes CVID was found in a total of 11 patients with deficiency of the inducible costimulator (ICOS; Warnatz et al., 2006; Takahashi et al., 2009). Only one of the original nine European patients presented with AI neutropenia, whereas AI manifestations were more prominent in the two Japanese patients presenting with (rheumatoid) arthritis, inflammatory bowel disease, interstitial pneumonitis, and psoriasis.

Finally, many reports have described reduced numbers of circulating regulatory T cells in CVID, especially affecting Freiburg Ia patients with reduced switched memory B cells and expansion of CD21^low^ B cells (see below; Fevang et al., 2007; Genre et al., 2009; Horn et al., 2009; Melo et al., 2009; Yu et al., 2009; Arumugakani et al., 2010; Mouillot et al., 2010). Several of the factors mentioned above, such as a CID-like phenotype with or without a disturbed TCR signal (Picard et al., 2009; Sauer et al., 2012), cytokine disturbance (Setoguchi et al., 2005), and even persistent CD4 lymphopenia itself (Matsuoka et al., 2010) might contribute to the reduction in regulatory T cells. Interestingly, even ICOS deficiency disturbs maintenance and function of regulatory T cells (Kornete et al., 2012), thus potentially rendering regulatory T cell deficiency a crucial element in AI dysregulation which is also common to different forms of immunodeficiency.

## DISTURBED B CELL HOMEOSTASIS IN AI-CVID

B cell homeostasis is also disturbed in CVID patients. Therefore, reduced switched memory B cell development and the expansion of activated CD21^low^ B cells are associated with the manifestation of AI-CVID (Warnatz et al., 2002; Sanchez-Ramon et al., 2008; Isnardi et al., 2010; Boileau et al., 2011). CD21^low^ B cells contain a high proportion of autoreactive clones (Rakhmanov et al., 2009; Isnardi et al., 2010) suggesting a disturbed selection of the B cell repertoire. This may involve defects in central selection for some (Isnardi et al., 2010), but not all patients (Rakhmanov et al., 2010). Several factors have been identified as interfering with B cell selection. Firstly, the signal strength of the BCR itself determines the outcome during selection (Khan, 2009). Several mouse models have demonstrated that alterations in the signaling machinery (Cornall and Goodnow, 1998; Wang and Clark, 2003) and the balance between co-stimulatory (Tedder et al., 1997) and inhibitory co-receptors (Cornall et al., 1998) determine the counter-selection of AI B cell clones. In CVID patients disturbed antigen receptor signaling was described and is discussed below.

Given the negative feedback loop of immune complexes on B cells and plasma cells via the inhibitory receptors (Seite et al., 2010; Baerenwaldt et al., 2011) it is intriguing to speculate as to whether low serum IgG by itself may contribute to antibody-mediated AI cytopenias as one of the first manifestations in AI-CVID. Signaling by FcγRIIB inhibits B cell activation and can even induce apoptosis in plasma cells (Xiang et al., 2007). Additionally, a lack of inhibition of monocytes/macrophages by FcγRIIB may foster overwhelming inflammatory responses and granuloma formation, a serious clinical problem seen in a subset of AI-CVID patients. Lupus-like disease in FcγRIIB-deficient C57BL/6 mice (Bolland and Ravetch, 2000) as well as the increased risk of SLE in homozygous carriers of the dysfunctional FcγRIIB I232T variant (Floto et al., 2005) clearly indicate a crucial role for this inhibitory receptor in the maintenance of humoral tolerance. This hypothesis is supported by the fact that in most CVID patients the initiation of immunoglobulin replacement leads to an amelioration of the bouts of AI-mediated cytopenias.

The other major factors, which contribute to B cell-mediated autoimmunity, are related to survival signals during selection (Cancro, 2004). For B cells, overexpression of B cell-activating factor (BAFF) causes increased survival of autoreactive B cells and overt autoimmunity (Mackay et al., 1999; Thien et al., 2004). It is noteworthy that most CVID patients present with elevated BAFF levels (Kreuzaler et al., 2012). Currently it is unknown whether elevated BAFF levels sustain the expansion of CD21^low^ B cells in CVID. The number of circulating CD21^low^ B cells increases in other AI diseases, such as SLE (Wehr et al., 2004), rheumatoid arthritis (Isnardi et al., 2010), and cryoglobulinemia (Terrier et al., 2011), supporting an association with autoimmunity. In contrast to SLE, where switched memory B cells are relatively expanded and active disease is associated with expansion of circulating plasmablasts (Dorner and Lipsky, 2004), AI-CVID has a more severe reduction in the number of switched memory B cells when compared to other CVID patients. This could represent a disturbed peripheral differentiation and selection. Increased autoimmunity associated with poor germinal center function has also been observed in deficiency of the AID (Hase et al., 2008), but no abnormalities of AID expression or function have been described in CVID at this point.

Of all the genetic mutations which are associated with CVID, AI manifestations are most common in TACI-deficiency [18/50 (36%) vs 112/490 (23%) in wt TACI CVID; Salzer et al., 2009]. In particular, heterozygous C104R mutations seem to effect a predisposition for autoimmunity (11/20 patients, 55%; Salzer et al., 2009). While partial TACI signals in a heterozygous state may contribute to the survival of autoreactive B cells, a formal proof of this hypothesis is still missing. AI manifestations including glomerulonephritis and vasculitis (interestingly with deposits of IgA) as well as AI thrombocytopenia (AI-TP) have also been described for CD19 and CD81 deficiency, and are possibly related to the disturbed antigen receptor signal in these patients (see also below; van Zelm et al., 2006, 2010; Vince et al., 2011). The other B cell-intrinsic genetic defects associated with CVID (BAFF-R, CD20, CD21) have not been reported with AI manifestations (Warnatz et al., 2009; Kuijpers et al., 2010; Thiel et al., 2011, but to date only single patients have been described for each defect, thus precluding definite conclusions.

In recent years, a B cell population producing IL10 has been described as regulatory B cells (Mauri and Bosma, 2012). Currently, nothing is known about their existence and function in CVID.

## DISTURBED ANTIGEN RECEPTOR SIGNAL IN AUTOIMMUNE CVID

Several mouse models of increased BCR signals demonstrate an increased prevalence of AI manifestations (Dorner and Lipsky, 2006). On the other hand, models of decreased TCR signaling can also represent a risk factor for autoimmunity (summarized in Liston et al., 2008). Decreased TCR signals are thought to interfere with negative selection either through a selective or a stronger impact on tolerogenic signals (Liston et al., 2008) thus potentially impairing the generation of regulatory T cells (Liston and Rudensky, 2007). In humans, ORAI (Feske et al., 2006) and Stim1 deficiency (Picard et al., 2009) need to be mentioned as prototypes of reduced antigen receptor signal strongly associated with the coincidence of immunodeficiency and autoimmunity in the affected patients. Also in B cells of the subgroup of CVID patients with an increased risk of AI manifestations, calcium signaling is reduced compared to other CVID patients and healthy controls (Foerster et al., 2010; van de Ven et al., 2011). The exact mechanism of the signaling defect and its potential interference with selection are unknown. In WAS, antigen receptor signaling is impaired due to mutations in the WAS protein (Zhang et al., 1999). Interestingly, WASP deficiency also leads to increased AI disease associated with decreased CD27^+^ memory B cells and increased CD21^low^ B cells (Park et al., 2005). Although WASP deficiency affects both T and B cell receptor signaling, B cell-intrinsic defects clearly contribute to autoimmunity in WAS (Recher et al., 2012). As indicated above, previous reports have found disturbed TCR-induced calcium signals (Fischer et al., 1996) in 40–50% of CVID patients but a link to immune dysregulation in the identified patients has not been established.

## ALTERED TYPE I INTERFERON SIGNAL IN AUTOIMMUNE CVID

Cytokines have been implicated in AI dysregulation. Type I IFNs are thought to be particularly important as (i) AI reactions are induced in patients after treatment with type I IFNs, (ii) the *IFN* signature is increased in patients with SLE, and (iii) some chronic viral infections are associated with autoimmunity (Hall and Rosen, 2010). The mechanisms are manifold and include induction of dendritic cell (DC) maturation and increased BAFF production, a positive feed back loop in toll-like-receptors (TLR) 7 and 9 signaling leading to class switched antibody production (Hall and Rosen, 2010).

Type I IFNs have not been well examined in CVID patients. There exists only a single report of increased type I IFN production in CVID patients (Strannegard et al., 1987); others have detected increased MxA expression as a marker of IFN exposure in leukocytes of only 2/13 CVID patients (Rump et al., 1995). So far no attempt to correlate in CVID IFN expression to AI manifestations has been made.

Type I IFN expression and the induction of AI reactions is closely linked to the activation of TLRs on plasmacytoid DCs (pDCs) and B cells (Green and Marshak-Rothstein, 2011). Different strains of AI prone mice rendered deficient in TLR7/9 or MyD88 expression produce dramatically fewer autoantibodies and develop less severe disease (Green and Marshak-Rothstein, 2011). Surprisingly, however, TLR9 deficiency in the presence of normal TLR7 function reduces only anti double-strain-DNA autoantibody levels, but not other autoantibodies and is associated with a more severe AI disease, suggesting a regulatory role of TLR9 for TLR7-mediated immune disease. In CVID patients, pDC and B cell responses to TLR7 and 9 ligands are impaired (Yu et al., 2012). Subanalysis of the reported data suggests that a subgroup of patients is more seriously affected by reduced TLR signaling. While the authors correlate the reduced function to increased infection susceptibility no correlation to autoimmunity is mentioned.

In summary, autoimmunity is a prominent clinical feature in CVID. Associated factors include disturbed B and T cell homeostasis and selection, altered antigen receptor signals, increased BAFF levels, and possibly altered TLR signaling. Pathogenic mechanisms, however, have not been identified yet on a molecular level. Further research needs to consider established mechanisms in other genetically defined immunodeficiency disorders to unravel the underlying immune dysregulation in CVID. Our improved knowledge will not only steer potential treatment strategies but also our concept of autoimmunity in general.

## Conflict of Interest Statement

The authors declare that the research was conducted in the absence of any commercial or financial relationships that could be construed as a potential conflict of interest.
